# Influence of Mg Doping on ZnO Nanoparticles for Enhanced Photocatalytic Evaluation and Antibacterial Analysis

**DOI:** 10.1186/s11671-018-2643-x

**Published:** 2018-08-03

**Authors:** K. Pradeev raj, K. Sadaiyandi, A. Kennedy, Suresh Sagadevan, Zaira Zaman Chowdhury, Mohd. Rafie Bin Johan, Fauziah Abdul Aziz, Rahman F. Rafique, R. Thamiz Selvi, R. Rathina bala

**Affiliations:** 1 Department of Physics, CSI College of Engineering, Ooty, 643215 India; 2 Research and Development Centre, Bharathiar University, Coimbatore, 641046 India; 3 Department of Physics, Government Arts College for Women, Nilakkottai, Dindigul 624202 India; 4 Centre for Nanotechnology, AMET University, Kanathur, Chennai, Tamil Nadu 602 105 India; 5 Nanotechnology and Catalysis Research Centre, University of Malaya, Kuala Lumpur, 50603 Malaysia; 6 Department of Physics, Center for Defence Foundation Studies, National Defence University of Malaysia, Kuala Lumpur, Malaysia; 7 Rutgers Cooperative Extension Water Resources Program, Rutgers, The State University of New Jersey, New Brunswick, USA; 8 Department of Chemistry, LRG Government Arts College for Women, Tiruppur, 641 604 India

**Keywords:** Mg-doped ZnO nanoparticles, Structural study, Optical study, Photocatalytic evaluation, Antibacterial study

## Abstract

In this research, a facile co-precipitation method was used to synthesize pure and Mg-doped ZnO nanoparticles (NPs). The structure, morphology, chemical composition, and optical and antibacterial activity of the synthesized nanoparticles (NPs) were studied with respect to pure and Mg-doped ZnO concentrations (0–7.5 molar (M) %). X-ray diffraction pattern confirmed the presence of crystalline, hexagonal wurtzite phase of ZnO. Scanning electron microscope (SEM) images revealed that pure and Mg-doped ZnO NPs were in the nanoscale regime with hexagonal crystalline morphology around 30–110 nm. Optical characterization of the sample revealed that the band gap energy (*E*_g_) decreased from 3.36 to 3.04 eV with an increase in Mg^2+^ doping concentration. Optical absorption spectrum of ZnO redshifted as the Mg concentration varied from 2.5 to 7.5 M. Photoluminescence (PL) spectra showed UV emission peak around 400 nm. Enhanced visible emission between 430 and 600 nm with Mg^2+^ doping indicated the defect density in ZnO by occupying Zn^2+^ vacancies with Mg^2+^ ions. Photocatalytic studies revealed that 7.5% Mg-doped ZnO NPs exhibited maximum degradation (78%) for Rhodamine B (RhB) dye under UV-Vis irradiation. Antibacterial studies were conducted using Gram-positive and Gram-negative bacteria. The results demonstrated that doping with Mg ions inside the ZnO matrix had enhanced the antibacterial activity against all types of bacteria and its performance was improved with successive increment in Mg ion concentration inside ZnO NPs.

## Background

Nanoparticles exhibit novel properties which depend on their size, shape, and morphology which enable them to interact with plants, animals, and microbes [1]. Nanoparticles of commercial importance are being synthesized directly from metal or metal salts, in the presence of some organic material or plant extract. The creepers and many other plants exude an organic material, probably a polysaccharide with some resin, which helps plants to climb vertically or through adventitious roots to produce nanoparticles of the trace elements present, so that they may be absorbed [2]. Nanosize inorganic compounds have shown remarkable antibacterial activity at very low concentration due to their high surface area to volume ratio and unique chemical and physical features [3]. In addition, these particles are also more stable at high temperature and pressure [4]. Some of them are recognized as non-toxic and even contain mineral elements which are vital for the human body [5]. It has been reported that the most antibacterial inorganic materials are metallic nanoparticles and metal oxide nanoparticles such as silver, gold, copper, titanium oxide, and zinc oxide [6, 7]. Zinc is an essential trace element for the human system without which many enzymes such as carbonic anhydrase, carboxypeptidase, and alcohol dehydrogenase become inactive, while the other two members, cadmium and mercury belonging to the same group of elements having the same electronic configuration, are toxic [8]. For biosynthesis of nanoparticles, different parts of a plant are used as they contain metabolites such as alkaloids, flavonoids, phenols, terpenoids, alcohols, sugars, and proteins which act as reducing agents to produce nanoparticles. They also act as capping agent and stabilizer for them. They are used in medicine, agriculture, and many other fields as well as technologies. The attention is therefore focused on all plant species which have either aroma or color in their leaves, flowers, or roots for the synthesis of nanoparticles because they all contain such chemicals as will reduce the metal ions to metal nanoparticles [9]. Recently, nanoparticles have started gaining interest because of their unique mechanical, optical, magnetic, electrical, and other properties [10]. These emergent characteristics make them a promising candidate for use in electronics, medicine, and other fields. It has been observed that nanomaterials have a greater surface to volume ratio compared with their conventional forms [11]. This is the reason why nanomaterials show greater chemical reactivity. Basically at nanoscale, the quantum effect is more pronounced to determine their end characteristics leading to novel optical, magnetic, and electrical behavior. Among the various metal oxide semiconductor nanostructures, ZnO nanostructures have attracted considerable interest because of their low cost, high chemical stability, and mass production [12]. ZnO research only makes up a reasonable bit of the current nano-look into; however, all factors considered it is one of the important materials which will presume an expanding part of the nanotechnology without bounds. ZnO is one of the most generally connected topical drugs ever. It is utilized in most sunscreens and discovers its way into numerous treatments for anguish and itching alleviation [13]. It will be seen on the function of photocatalysis, essentially on ZnO and doped ZnO; there is a large cluster of potential chemical, photochemical, and electrochemical responses that can happen on the photocatalyst surface [14]. ZnO has won much consideration in the degradation and total mineralization of environmental contaminations [15]. The best-preferred point of view of ZnO is the capacity to attract an extensive variety of solar-powered light range and more light quanta than some semiconducting metal oxides. ZnO has developed as the main material and a proficient and promising candidate in green ecology management structure due to its novel attributes [16].

Basically two factors, namely surface area and surface defects, are the most important variables to determine the photocatalytic activity of semiconductor metal oxides. Due to its high surface activity, crystalline nature, morphological features, and texture; ZnO nanoparticles are considered as the most favorable catalyst for the degradation of organic pollutants [17]. Recent literature reported that Mg-doped ZnO nanostructures can exhibit excellent properties for device application [18]. Investigation on doping Group II elements with ZnO showed that the dopants can alter the band gap energy (*E*_g_) with an increase in the UV-Visible luminescence intensity [19].

Doping of Mg into ZnO is expected to modify the absorption, physical, and chemical properties of ZnO [20]. Metal ion-doped ZnO nanostructures are the most promising catalyst for the degradation of various pollutants because of its enhancement in its optical properties [21]. Different types of infectious diseases caused by bacteria pose a severe menace towards the public health worldwide. To enhance the antibacterial activity of ZnO, different types of physiochemical properties such as particle size, crystallinity index, and optical properties should be modified by doping with metal or non-metal [22].

By inducing more defects over the surface of ZnO, the optical adsorption properties can be enhanced. Basically minute amounts of dopants are sufficient to act as donors or acceptors inside the semiconductor crystal lattice which will significantly alter the properties of the semiconductor up to a greater extent. The size quantization makes variation in the energy gap between the conduction band electrons and valence band holes which result in change in optical properties of the doped metal oxide nanostructures [23]. Earlier literature reported that ZnO nanoparticles can resist bacterium and they have the ability to shield ultraviolet radiations [24]. Zinc oxide NPs have the ability to disrupt the gram-negative cell membrane structure of *Escherichia coli* [25, 26]. It was reported also that nanoparticles with a positive charge could bind the gram-negative cell membrane using electrostatic attraction [27]. ZnO NPs doped with different metal ions were evaluated against *E. coli*, and *Staphylococcus aureus* showed the antibacterial activity increasing with crystallite size [28].Various physical and chemical techniques have been adopted by researchers to synthesize pure and doped ZnO NPs like the vapor transport process [29], spray pyrolysis [30], thermal decomposition [31], electrochemical method [32], sol-gel method [33], hydrolysis [34], chemical precipitation [35], and hydrothermal method [36] in order to tailor its morphology and size. Among all these methods, co-precipitation method is relatively simple and inexpensive. Furthermore, it can give high yield at room temperature for the synthesis of pure and doped ZnO NPs [37].

Here, we have investigated the preparation and characterization of ZnO nanoparticles with different concentrations of Mg dopants by using a simple chemical co-precipitation method. The effects of Mg^2+^ ion concentration inside the ZnO lattice have been evaluated in terms of structural, morphological, optical, and photocatalytic studies. Further, the effect of Mg^2+^ ions on the antibacterial activity were studied against (Gram-positive and Gram-negative) *S. aureus*, *E. coli*, and *Proteus* cultures.

## Methods

**Fig. 1:**
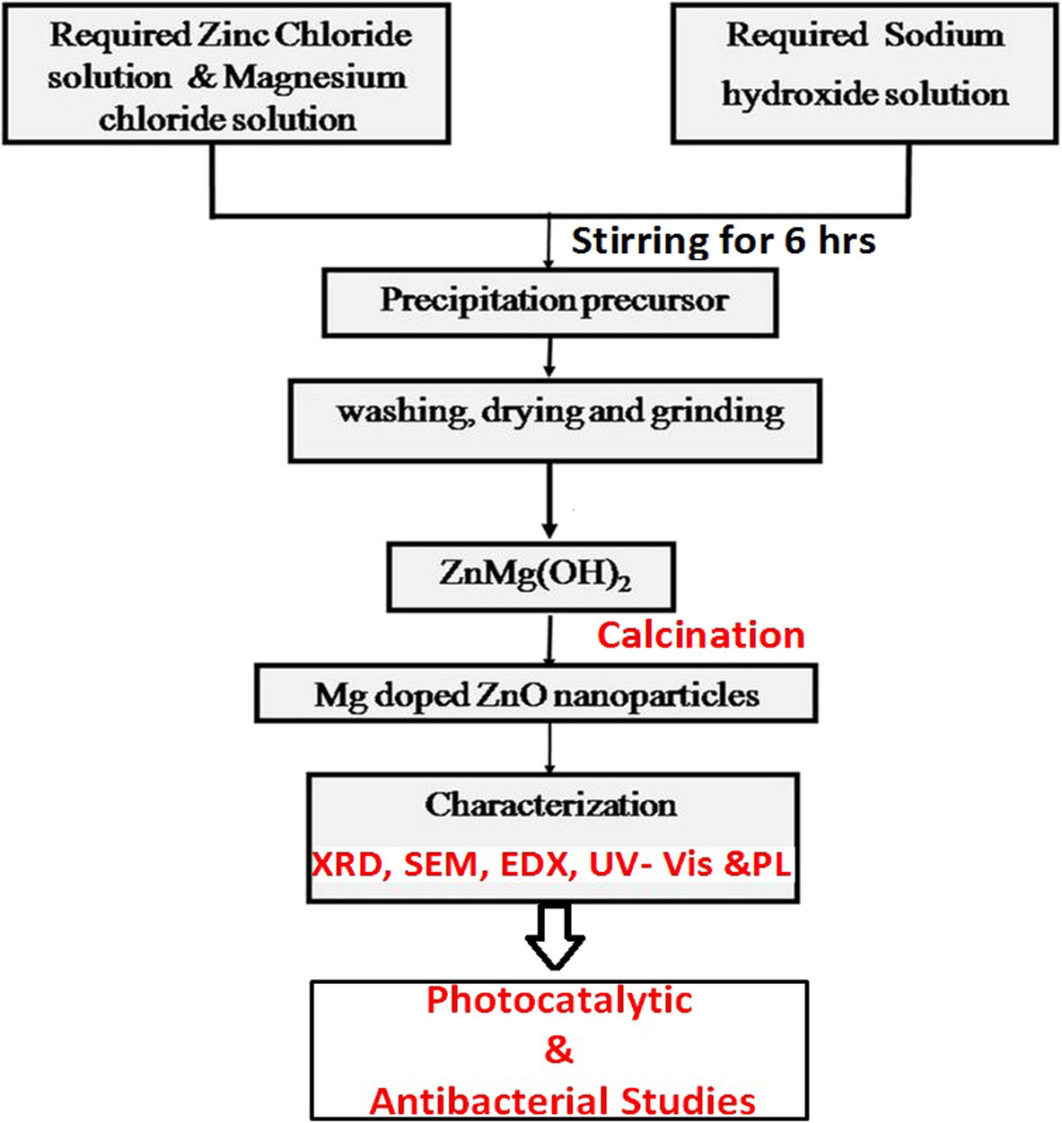
Flow chart describing the synthesis of Mg-doped ZnO NPs

All the reagents of analytical grade were purchased from Sigma-Aldrich and used as received without further purification. The flow chart (Fig. 1) describes the preparation method of the pure and Mg-doped ZnO NPs. In this method, 1 M of sodium hydroxide (NaOH) solution is added to 1 M of zinc chloride (ZnCl_2_) solution. The final solution is kept under constant stirring for 6 h. The alkaline solution of sodium hydroxide helps in the precipitation of the transition metal hydroxides. It accelerates the reduction process, thereby causing the formation of ZnO and Mg-doped ZnO nanoparticles. Furthermore, sodium hydroxide converts ZnCl_2_ to Zn(OH)_2_ which after heating yields ZnO nanoparticles. After the precipitation, the beaker is taken out and sufficient time is given to settle the final product. The product obtained is filtered and washed several times with deionized water and acetone. Finally, the samples are dried at 100 °C for 5 h and then converted to a fine powder by grinding in an agate mortar. The powders thus obtained are calcined at 300 °C for 4 h to yield nano-sized ZnO particles.

To synthesize Mg-doped ZnO, molar ratios of ZnCl_2_ and MgCl_2_ were measured as Zn_1 − *x*_ Mg _*x*_O (where *x* = 0.025, 0.050, and 0.075) and the same procedures were repeated. The chemical equation for the synthesis of pure and Mg-doped ZnO are shown in Eqs. (1–4)

1 $$ {\mathrm{ZnCl}}_2+2\mathrm{NaOH}\to \mathrm{Zn}{\left(\mathrm{OH}\right)}_2+2\mathrm{NaCl}\left(\mathrm{aq}\right) $$

2 $$ \mathrm{Zn}{\left(\mathrm{OH}\right)}_2\to \mathrm{Sintering}\to \mathrm{Mg}\hbox{-} \mathrm{doped}\ \mathrm{ZnO}+{\mathrm{H}}_2\mathrm{O} $$

3 $$ {\mathrm{MgCl}}_2+{\mathrm{ZnCl}}_2+2\mathrm{NaOH}\to \mathrm{ZnMg}{\left(\mathrm{OH}\right)}_2+2\mathrm{NaCl}\left(\mathrm{aq}\right) $$

4 $$ \mathrm{ZnMg}{\left(\mathrm{OH}\right)}_2\to \mathrm{Sintering}\to \mathrm{Mg}\hbox{-} \mathrm{doped}\ \mathrm{ZnO}+{\mathrm{H}}_2\mathrm{O} $$

In the electrochemical series, Mg is more reactive than Zn and hence it undergoes reduction to occupy the Zn lattice.

### Characterization

The crystal structures of the samples were investigated by X-ray diffraction (XRD) Bruker D8 advanced X-ray diffractometer) with Cukα radiation (*λ* = 1.54 Å) and surface morphology features were studied by field emission scanning electron microscope (FESEM, ZEISS). Optical absorption spectra of the samples were recorded with a double beam UV-Visible spectrophotometer using Hitachi U-3900H in the range 200–1200 nm. Photoluminescence (PL) emission studies were carried out by means of a spectrometer (JOB HR800 IN Yoon Horbe) using a He-Cd laser source with a wavelength of 325 nm. The antibacterial activity of the synthesized samples was tested towards different organisms by agar disc diffusion technique.

### Measurement of Photocatalytic Activity

Experiments were carried out in a photocatalytic quartz reactor having a capacity of 150 ml. The reactor had facilities for water circulation to ensure a constant temperature. The UV irradiation was carried out by using 125 W (311 nm) medium pressure Hg arc lamp (SAIC, INDIA). One hundred fifty milliliters of desired initial concentration (20 ppm) of RhB dye solution was mixed with a fixed amount ZnO NPs (50 mg/L) at natural pH (6.2). The solution was placed under UV illumination and was magnetically stirred. The sample from the photoreactor was withdrawn at different time intervals and centrifuged. The supernatants were analyzed for its absorption maximum (554 nm) using UV-Vis spectrophotometer. Similar procedure was adopted for Mg dopants (2.5, 5, and 7.5%)-ZnO NPs using RhB dye solution. Percentage of RhB degraded by the catalyst surface was calculated from the following equation:

5 $$ \mathrm{Percentage}\ \mathrm{of}\ \mathrm{degradation}=\left({C}_0-{C}_t\right)/{C}_0\times 100\% $$

where *C*_0_ represents the initial time of absorption and *C*_t_ represents the absorption after various intervals of time (0, 30, 60, 90, and 120 min).

### Antibacterial Studies

*E. coli* (Gram-negative), *S. aureus* (Gram-positive), and *Proteus* (Gram-negative strains) were maintained at 4 °C on broth media before use. Nutrient agar medium was prepared and sterilized at 121 °C for 15 min. Twenty-five milliliters of nutrient agar was poured into sterile Petri dishes and setting was allowed. In each Petri dish was spread 0.2 ml of different bacterial species (*E. coli*, *S. aureus*, and *Proteus*). A disc was prepared and placed in the plates with the help of a sterile loop, and two discs per plates were made into the set agar containing the bacterial culture.

## Results and Discussion

### Structural Studies

**Fig. 2:**
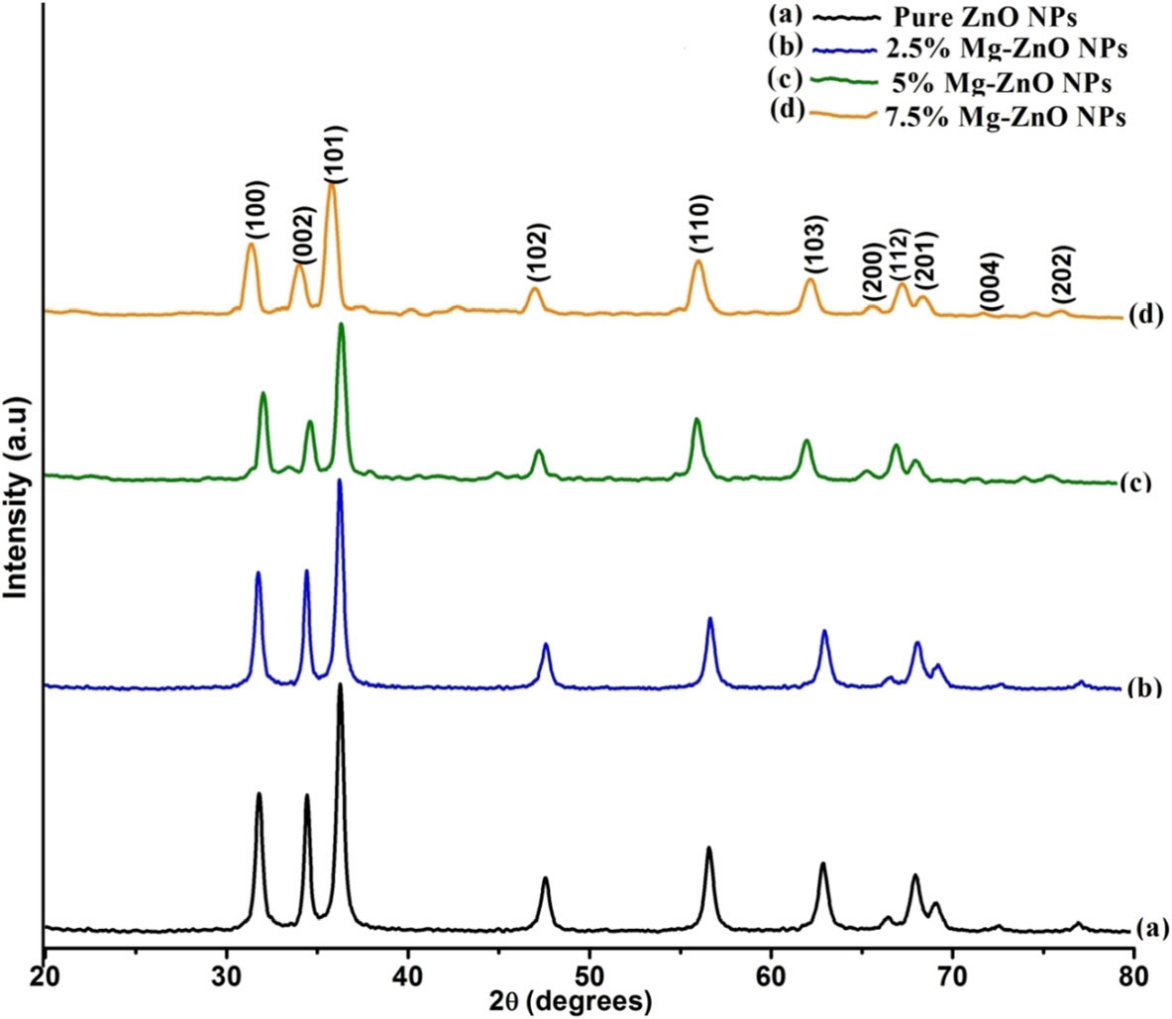
XRD pattern for pure and Mg-ZnO NPs. **a** Pure ZnO NPs. **b** 2.5% Mg-ZnO NPs. **c** 5% Mg- ZnO NPs. **d** 7.5% Mg- ZnO NPs

Figure 2 illustrates the X-ray diffraction (XRD) patterns for the pure and Mg-doped ZnO samples. In the figure, seven major peaks are seen at 31.8°, 34.5°, 36.3°, 47.5°, 56.7°, 62.9°, and 68° which can be consigned to diffraction from (100), (002), (101), (102), (110), (103), and (112) planes respectively for a lattice constant of a = b = 3.24 Å and c = 5.2066 Å [38]. The XRD pattern clearly reflects the presence of the hexagonal wurtzite phase crystal structure for pure ZnO nanoparticles (JCPDS: 36-1451) [39]. Also from the diffraction, it is noted that no further secondary phases are observed with Mg dopant into ZnO crystal lattice and no significant changes are observed in the XRD pattern of the Mg-doped ZnO NPs. It is also noted that the intensity of the XRD peak decreases with increase in Mg doping concentration (shown in Fig. 2a–d) which confirms the slender loss in their crystallinity due to distortion of lattice. Due to Mg ions doping inside the periodic crystal lattice of ZnO, a small amount of strain is persuaded. This results in the swap of the lattice which consecutively leads to change the regularity of crystal. However, very careful inferences indicate that the peak position shifts towards the lower angle values as observed with higher doping of Mg into ZnO matrix. Especially for the peak located at (101) plane 35°.84, it is found that it does shift towards lower value with increase in doping concentration, which can be attributed to the replacement of Zn^2+^ ions by Mg^2+^ ions [40]. It is well reported in the literature that the lattice characteristics of the host materials get changed due to the incorporation of dopant materials. This happens due to their variance in the atomic radii. Furthermore, the dopant ions may replace Zn ions in the host lattice (Mg ions) [41]. Thus the basic structure of ZnO NPs is unaltered and they retain their original wurtzite structure. This indicates that most of the Mg^2+^ ions go into the lattice as substitution ions to replace the Zn^2+^ ions and do not enter the void spaces. Since the ionic radius of the substituted Mg^2+^ (*R*_Mg_^2+^ = 0.057 nm is 0.57 Å) is smaller than that of Zn^2+^ (*R*_Zn_^2+^ = 0.06 nm is 0.60 Å) [42], it is observed that the shift corresponds to a small amount of lattice strain on the account of Mg^2+^ into ZnO environment.

The mean crystallite size is calculated by using Scherrer formula [43].

6 $$ d=\frac{0.89\lambda }{\beta \cos \theta } $$

where *λ* is the wavelength of the radiation (1.54056 Å), *β* is the full width at half maximum intensity, and *θ* is the diffraction angle. From the calculated values, it is observed that the mean crystallite size increases with an increase in Mg doping concentration (Table 1).

**Table 1:** Lattice parameters of pure and Mg-ZnO NPs

Sample	Crystallite size (D)	Lattice parameter			Atomic packing fraction (APF) %	Volume (nm^3^)	Strain
	nm	a (Å)	c (Å)	c/a ratio			No unit
ZnO NPs	24	3.2621	5.2256	1.603	75.84	48.21	1.995 × 10^− 3^
2.5% Mg-ZnO NPs	42	3.2618	5.2203	1.602	75.65	48.32	1.552 × 10^− 3^
5% Mg-ZnO NPs	61	3.2585	5.2181	1.602	75.47	48.41	1.201 × 10^−3^
7.5% Mg-ZnO NPs	90	3.2541	5.1999	1.599	75.22	48.50	1.043 × 10^−3^

### Effect of Doping on Lattice Parameters

The crystallite size, lattice parameters, atomic packing fraction (APF), lattice strain, and volume show the physical properties of pure and doped ZnO NPs [44]. For a wurtzite phase, the lattice parameters are calculated by using the Eq. (7–9) where, a = b, and c are the lattice parameters, *d*_*hkl*_ is the interplanar distance corresponding to its Miller indices (hkl).

7 $$ \frac{1}{d_{hkl}}=\frac{\left({h}^2+{k}^2\right)}{a^2}+\frac{l^2}{c^2} $$

8 $$ a=\frac{\lambda }{\sqrt{3\sin {\theta}_{100}}} $$

9 $$ c=\frac{\lambda }{\sin {\theta}_{002}} $$

$$ D=\frac{1}{A.P.F} $$

The calculated lattice parameters are listed in Table 1. It is observed from Table 1 that there is an alteration in its lattice parameter values as Mg^2+^ ion substitutes Zn^2+^ ion in the lattice. As the doping concentration increases, the dopant atom incorporated occupies the substitutional lattice site. It is also observed from Table 1 that the crystallite size (D) varies inversely with the atomic packing fraction (APF) as shown in Eq. (10).

Strain induced is calculated using Eq. (10).

10 $$ \varepsilon ={\beta}_{hkl}\cos \theta /4 $$

Furthermore, it is noted that there is a decrease in its lattice strain due to Mg^2+^ ions doping inside the ZnO matrix (Table 1), which causes the local distortion of the crystal structure. This is evident and noted earlier also in the literature for the difference in their atomic radii as well as in their doping concentration [45].

### Field Emission Scanning Electron Microscope (FESEM) and EDS Analysis

**Fig. 3:**
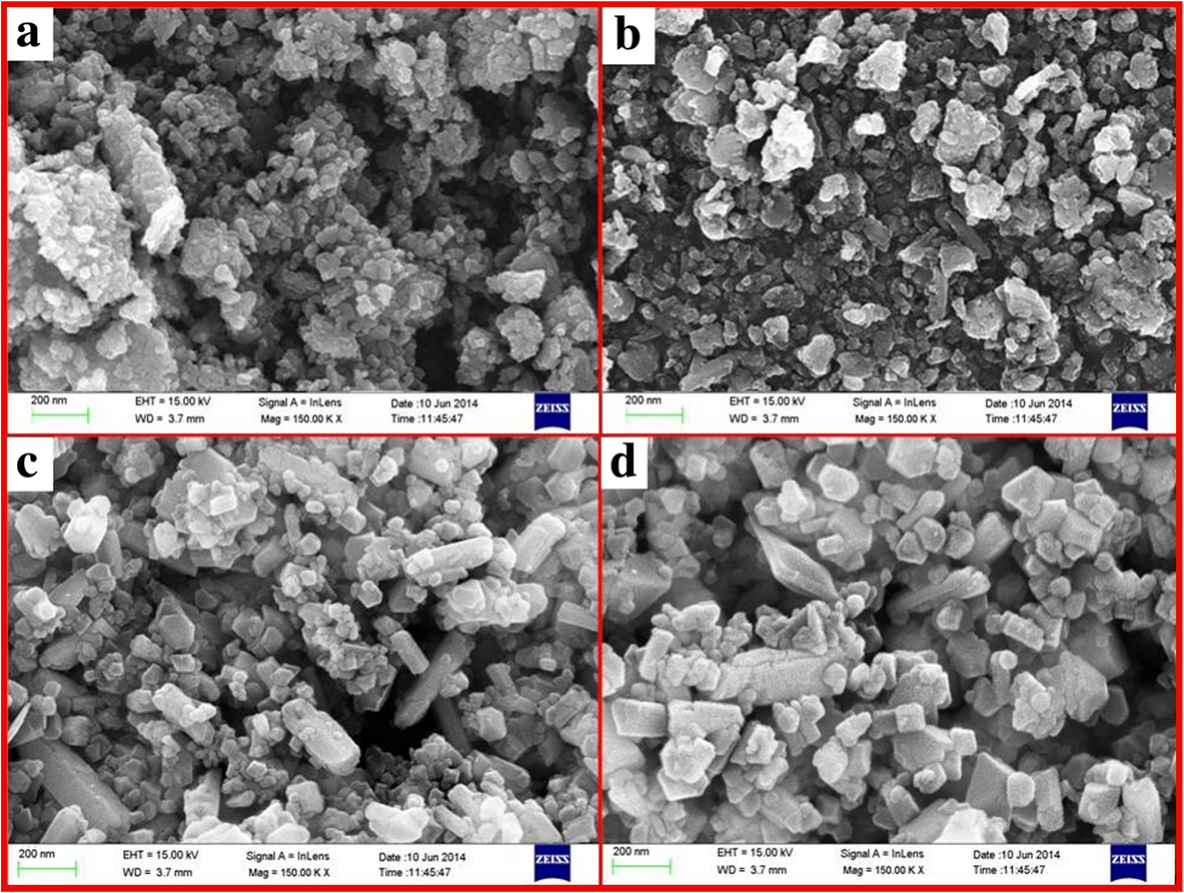
FESEM photographs of pure and Mg-doped ZnO NPs. **a** Pure ZnO NPs. **b** 2.5% Mg-ZnO NPs. **c** 5% Mg-ZnO NPs. **d** 7.5% Mg- ZnO NPs

Figure 3a–d illustrates the morphology of Mg-doped ZnO NPs at different Mg molar concentrations. From the FESEM images, it is observed that most of the grains fall in the nanoscale regime. It is also noted that the particles get aggregated on their surface. Aggregation of particles on the surface might have originated from the high surface energy of the synthesized NPs [46]. It is interesting to note that for a doping concentration of 5 M % and 7.5 M % some hexagonal crystal-shaped and well-distributed nanostructured grains are observed indicating the influence of higher doping Mg on the surface of ZnO matrix. With increased concentration of Mg ions inside the ZnO matrix, the grain size of the final particles was increasing from 30 to 110 nm. Figure 3c, d is in accordance with the crystallite size obtained by the XRD analysis.

**Fig. 4:**
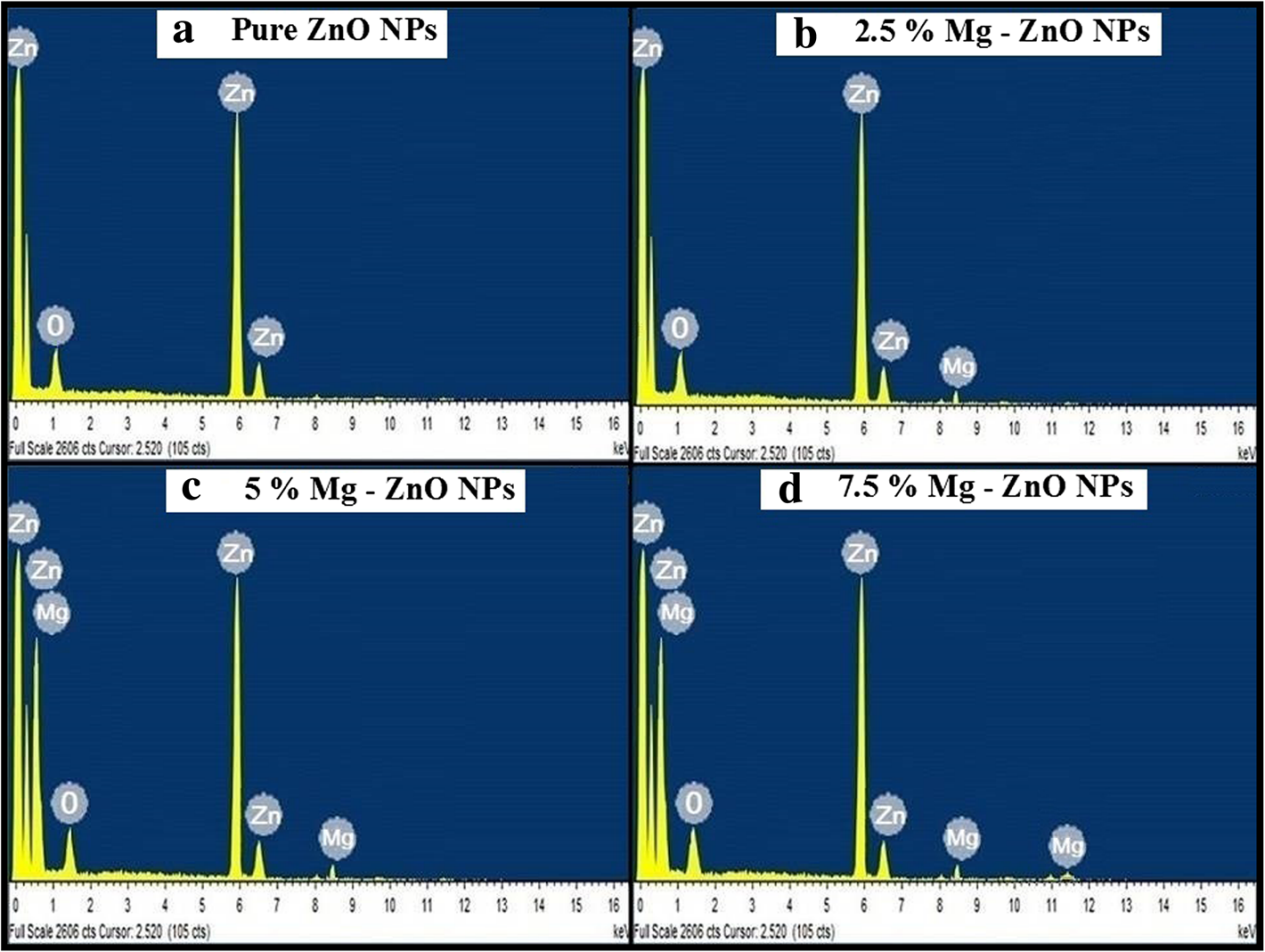
Energy-dispersive X-ray (EDS) spectra. **a** Pure ZnO NPs. **b** 2.5% Mg-ZnO NPs. **c** 5% Mg-ZnO NPs. **d** 7.5% Mg-ZnO NPs

Figure 4a–d displays the chemical compositional analysis of pure and Mg-doped ZnO nanoparticles carried out using EDS. From the obtained EDS spectra, the presence of various elements such as Zn, Mg, and O are observed. Figure 4c, d clearly shows the intensity of Mg slightly increasing with the injecting of Mg into ZnO environment. The incorporation of Mg ions had a significant effect on the structural and optical properties. It was also concluded from the EDS spectrum that no other foreign elements were present in the synthesized samples.

### Optical Studies

**Fig. 5:**
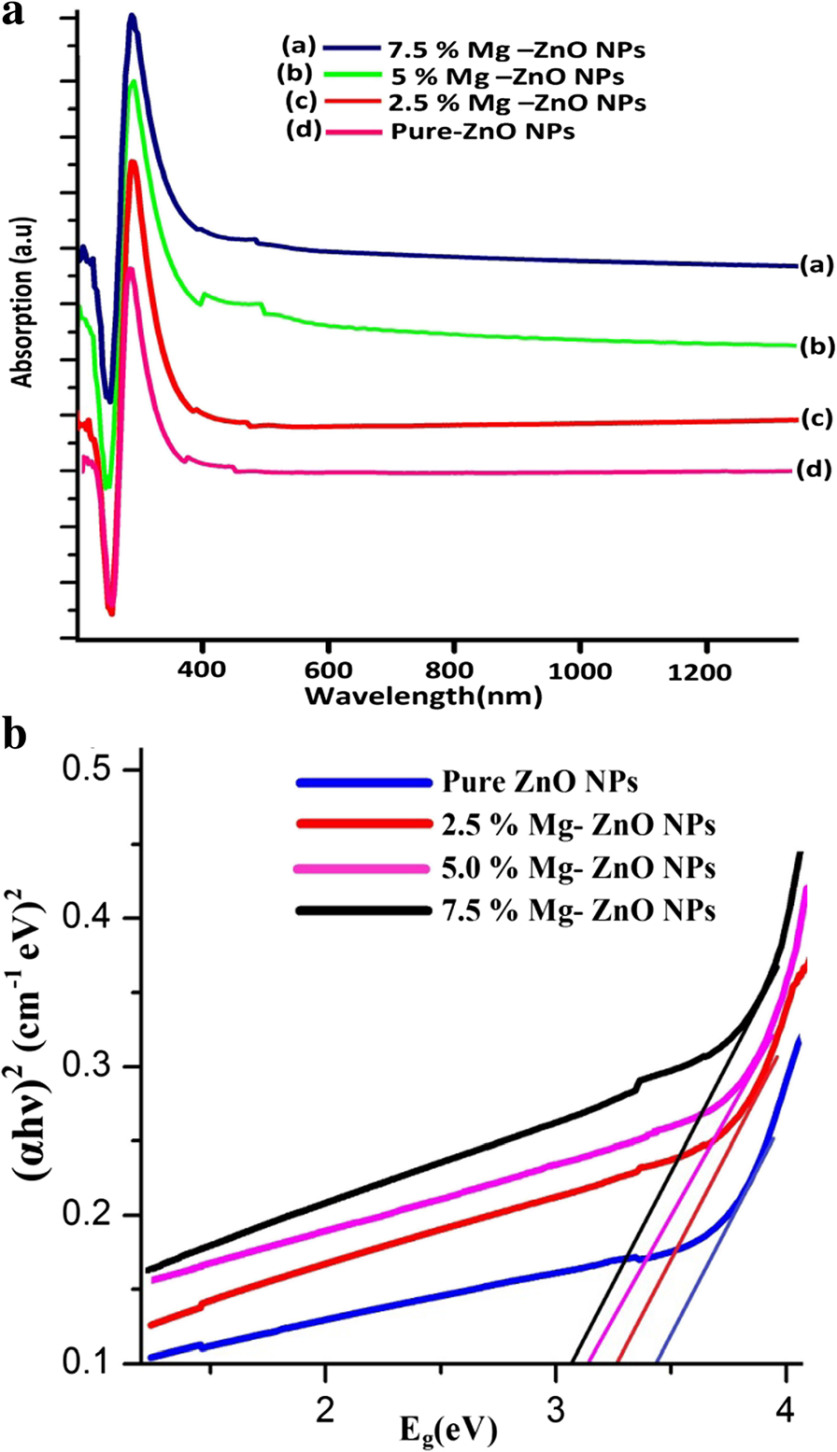
**a** Optical absorption and **b** band gap energy (*E*_g_) for pure and Mg-doped ZnO NPs

The UV-Vis absorption spectra of pure and Mg-doped ZnO NPs as a function of wavelength for the range of 200 to 1200 nm are illustrated by Fig. 5a. From the figure, it is noted that the absorption peak increases with the doping concentration. The increase in absorbance may be due to various factors like particle size, oxygen deficiency, and defects in grain structure [47]. The strong absorbance is found for the wavelength below 380 nm for Mg-doped ZnO nanoparticles while a very low absorbance is observed in the visible region as observed from Fig. 5a. This is attributed to greater absorption of incident photon energy by the molecules present in the lower energy state getting excited to the higher energy levels.

It is observed that the absorption edge of Mg-doped ZnO NPs is shifted to the longer wavelength (redshift) as Mg content is changed from 2.5 to 7.5 M %. This might be due to the small amount of lattice strain present in the sample as a result of Mg dopant towards ZnO. This redshift behavior is expected to decrease in its band gap (*E*_g_) value. The optical bandgap (*E*_g_) is determined from a Tauc-plot from the following relation (11).

11 $$ \alpha =\frac{A{\left( h\nu -{E}_{\mathrm{g}}\right)}^{1/2}}{hv} $$

where *α* is absorption coefficient, *h* is Plank’s constant, *ν* is the frequency of light radiation, and *E*_g_ is the band gap energy, where “*n*” takes the value of ½ for allowed direct transition [48]. Plots of (αhν) ^2^ versus (hν) are made for pure and Mg-doped ZnO NPs. The band gap energy (*E*_g_) is obtained from the extrapolation of the linear portions of the plots onto the *x*-axis.

From Fig. 5b, it is found that the band gap energy (*E*_g_) for pure ZnO NPs is around 3.36 eV and decreases with Mg dopant (3.36 to 3.04 eV). The band gap is decreased due to strong quantum confinements and enhancement in their surface area to volume ratio [49]. Enhancement of redshift and decrease in band gap energy (*E*_g_) confirm the presence of Mg^2+^ inside the Zn^2+^ site of the ZnO lattice.

### Photoluminescence Studies

**Fig. 6:**
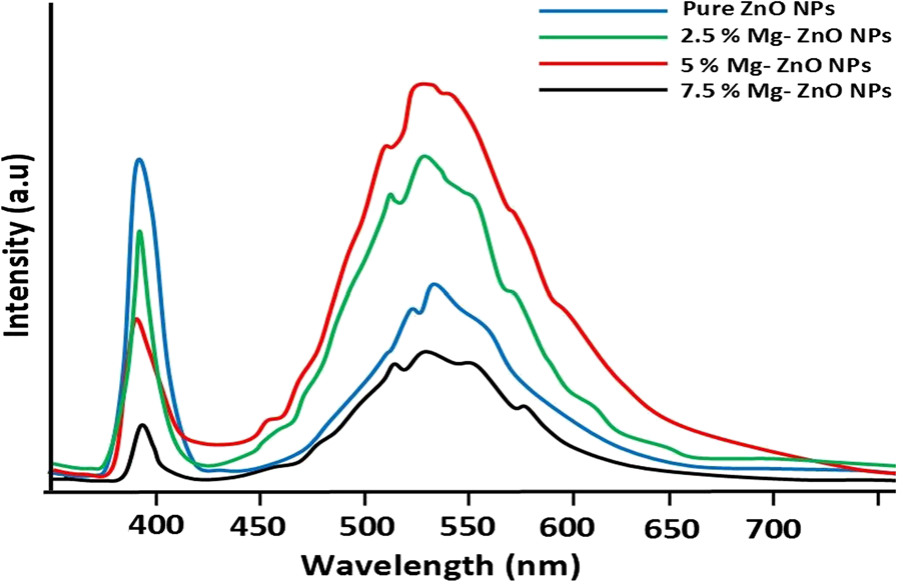
Photoluminescence spectrum of pure and Mg-ZnO NPs

Figure 6 illustrates the photoluminescence spectra for the pure and Mg^2+^-doped ZnO NPs at the wavelength of 325 nm. A relatively sturdy UV emission band around 400 nm and broad bands at 450 to 620 nm are observed in the visible spectrum region. Strong UV emission is attributed to the radiative recombination of excitons (exciton emission) [50]. The origin of the broad visible emission band at 450 to 620 nm is due to the surface anion vacancies [51]. This may be due to the tunneling of surface-bound electrons through pre-existing trapped holes [52]. It is also observed that the intensity of emission bands observed at 390 and 525 nm is decreased with higher doping of Mg content (7.5%). Higher doping percentages inside the ZnO NPs are preventing the recombination of photo-generated electrons and holes. Moreover, Mg (7.5%) ions produce additional active defect sites inside the ZnO lattice resulting in further visible light adsorption through these active defect sites [53].

### Photocatalytic Studies

**Fig. 7:**
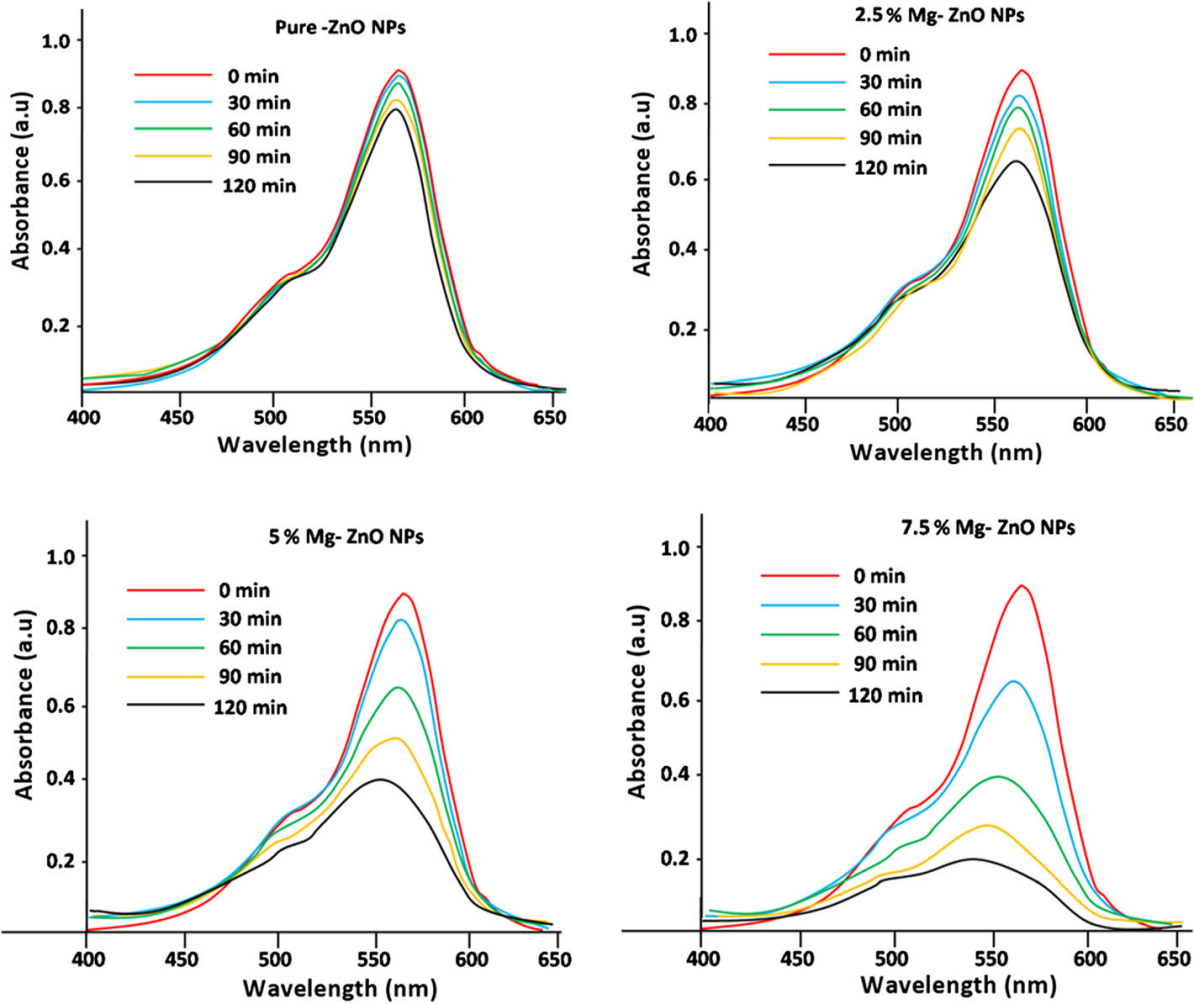
Absorption spectral decrement of Rhodamine B dye aqueous solution degraded from (0–120 min)

**Fig. 8:**
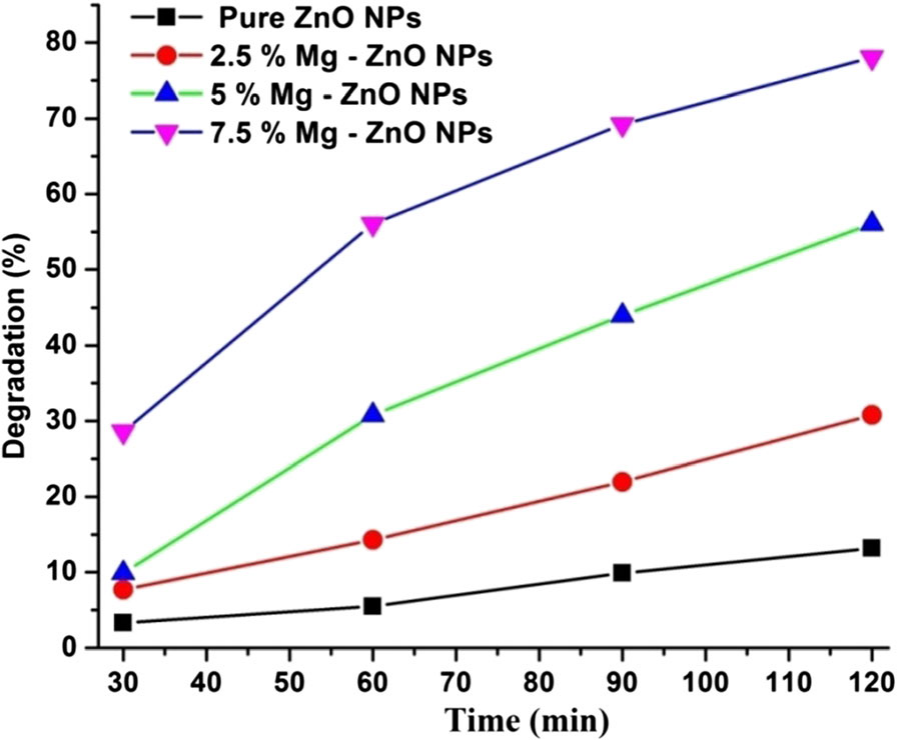
Photodegradation of Rhodamine B under pure and IZ-NPs

The photocatalytic degradation study of pure and Mg-doped ZnO with Rhodamine B (Rh B) dye solution was studied under different intervals of time (0–120 min). The optical absorption spectra of RhB dye solution at different time intervals (0–120 min) were recorded and the same is illustrated by Fig. 7. It is observed that with the lapse of time the peak height decreases indicating greater degradation of Rhodamine B due to the photocatalytic activity of ZnO. A negligible amount of the dye has been degraded using pure ZnO after 120 min, whereas the 7.5% Mg-doped sample showed higher degradation efficiency. This is anticipated due to presence of defects and oxygen vacancies created by Mg doping inside the ZnO matrix [54]. Figure 8 shows the percentage of degradation for pure and Mg-doped ZnO NPs. It is observed that 7.5% Mg-doped ZnO has exhibited a maximum degradation of 78% compared with the other doping concentrations (Table 2). It is also noted that a higher Mg (10% or more) doping concentration into ZnO will reduce the photocatalytic activity. This is understandable due to the physical defects as well as the increased oxidation states of cations. This phenomenon was observed earlier in the literature which described that excess cations produced during the doping process will act as trapping sites for the holes and electrons. Subsequently, this will stimulate the recombination of photo-generated charged species. This gradually impedes the generation of •OH (hydroxyl) and O•_2_^**−**^ (oxygen) superoxide radicals. This phenomenon will reduce the photocatalytic activity. Similar results have been reported by Lee et al. [55] and Yousefi et al. [56]. Further, in our co-precipitation technique, the thermodynamic solubility is less for higher doping concentration of Mg into ZnO. Similar to such results have been reported by Javed Iqbal et al [57].

**Table 2:** Catalytic degradation with band gap energy:(pure and Mg-doped ZnO NPs)

Sample	Band gap (*E*_g_) eV	Degradation (%) at 120 min	Degradation rate constant (k)
ZnO NPs	3.36	13.18	1.09 × 10^−3^
2.5% Mg-ZnO NPs	3.27	30.76	2.76 × 10^− 3^
5% Mg-ZnO NPs	3.13	56.04	5.72 × 10^− 3^
7.5% Mg-ZnO NPs	3.04	78.02	1.26 × 10^−2^

It seems that Mg-doped ZnO NPs act similar to electron sink, which consequently can enhance significantly the separation of the photo-generated electron−hole pairs and inhibit their recombination resulting in improved photocatalytic activity [58].

**Fig. 9:**
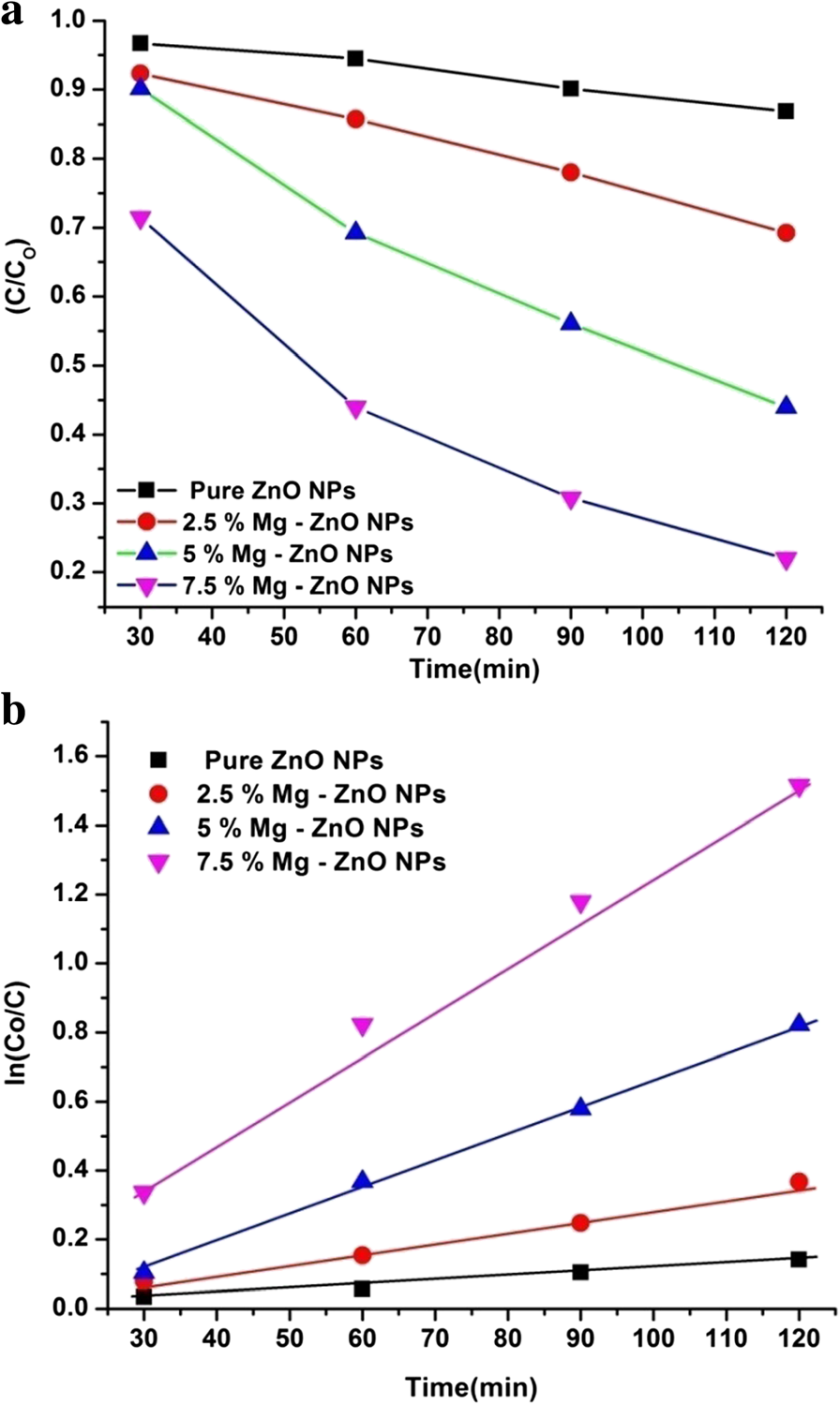
(**a**) Evolution of the relative concentration of RhB as a function of the time for pure and Mg-ZnO NPs (**b**) The reaction kinetics of RhB dye degradation for pure and Mg-ZnO NPs

The reaction kinetics can be observed by plotting linear curves for the concentration ratio, ln(C/C_o_), against the irradiation time “*t*”. From the graph (Fig. 9a), it is evidently visible that the existence of Mg ions from 2.5 to 7.5% inside the ZnO matrix has in fact activated the photocatalytic process. From Fig. 9b, the RhB degradation rate constant k was evaluated and it was 1.09 × 10^− 3^, 2.76 × 10^− 3^, 5.72 × 10^− 3^, and 1.26 × 10^− 2^ and for the pure ZnO NPs, 2.5% Mg-ZnO NPs, 2.5% Mg-ZnO NPs, and 2.5% Mg-ZnO NPs, respectively. Among them, the 7.5% Mg-ZnO NPs have exhibited the highest degradation rate constant (k) value, which has quite significantly increased compared with that of pure ZnO NPs (Table 2). The findings of this photocatalytic experiment clearly reveal that the doping of Mg ions up to a certain limit can effectively enhance the photocatalytic activity of ZnO photocatalyst.

The reason behind the enhanced photocatalytic activity for Mg-doped ZnO NPs is enlarged surface area with the presence of surface oxygen vacancies [59]. The photocatalytic mechanism of semiconductor materials proceeds through the formation of electron–hole pair (e^−^, h^+^) along with the subsequent separation as well as the recombination of electrons and holes [60]. Photocatalytic activity for pure ZnO is attributed both to the donor states caused by a large number of defect sites such as oxygen vacancies and interstitial zinc atoms and to the acceptor states which arise from zinc vacancies and interstitial oxygen atoms [61]. But for Mg-doped ZnO NPs for the degradation of RhB under UV-Visible irradiation, initially electron–hole pairs are created and then the species such as •OH and •O^− 2^ are formed as shown in the equation.

12 $$ ZnO+ h\nu \to ZnO\left({e}_{CB}+{h}_{VB}\right) $$

The photo-induced electrons are easily trapped by electronic acceptors like adsorbed (O_2_), in order to produce a superoxide radical anion (O•^− 2^) Eq. (13)

13 $$ {e}_{CB}+{O}_2\to {O}_2^{\bullet -} $$

Further, the photo-induced holes are easily trapped by negative OH^−^ ions to errand the production of hydroxyl radical species (OH•) Eq. (14)

14 $$ {OH}^{-}+{h}^{+}\to {OH}^{\bullet } $$

Thus produced OH^−^ radical and superoxide radical anion will carry out the total photocatalytic reaction. However, •OH radical is a particularly strong oxidant which can cause fractional or complete mineralization of organic molecules. The high oxidative potential of the hole in the valence band causes the oxidation of organic compounds to form some reactive intermediates [62] as shown by Eq. (15–16).

15 $$ {O}_2^{\bullet -}+\mathrm{RhB}\ \mathrm{degradation}\ \mathrm{products}+{CO}_2+{H}_2O $$

16 $$ {OH}^{\bullet }+\mathrm{RhB}\ \mathrm{degradation}\ \mathrm{products}+{CO}_2+{H}_2O $$

Thus, it is necessary to prevent the recombination of electron–hole pairs to have better photocatalytic activity of semiconductor based NPs. Controlled doping of Mg over the ZnO NPs up to a certain limit can enhance the photocatalytic activities. All the Mg-doped ZnO NPs show a significant enhancement of the photo-degradation of RhB dye compared with the pure ZnO NPs. In this research, 7.5% Mg-doped ZnO NPs show better photocatalytic properties after 120 min compared with pure ZnO sample. This might be due to the change in their particle size and band gap effects [63].

### Antibacterial Studies

**Fig. 10:**
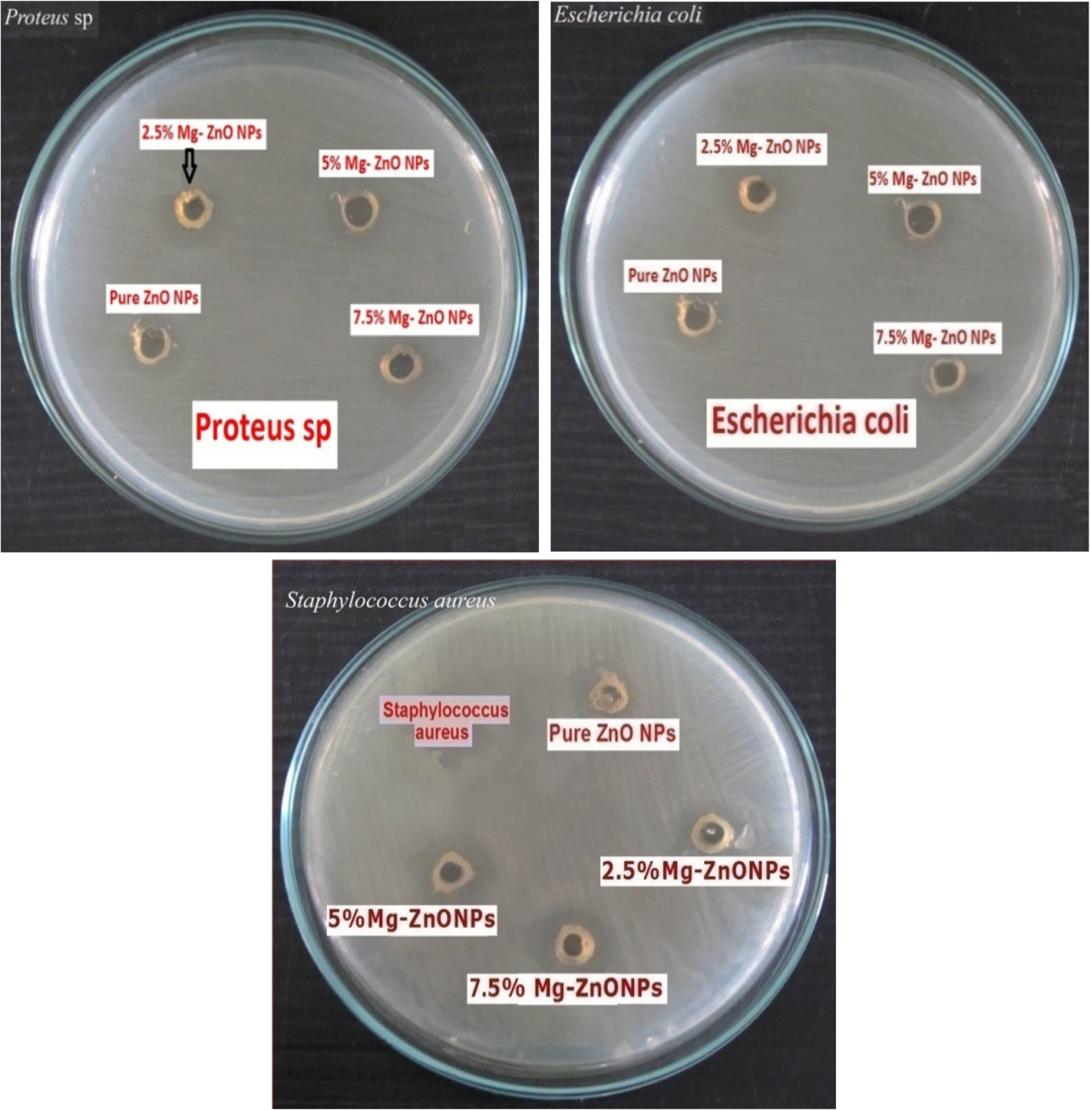
Zones of Inhibition of ZnO and Mg-doped ZnO NPs against the given bacteria

The zone of inhibition by using Mg-doped ZnO NPs for *E. coli* (Gram-negative), *S. aureus* (Gram-positive bacteria), and *Proteus* (Gram-negative strains) is displayed by Fig. 10. It was carried out using disc diffusion method to observe their ability as a potential antimicrobial agent. The prepared NPs were highly reactive due to their high surface to volume ratio. From Fig. 10, it is clear that the Mg^2+^-doped ZnO NPs inhibit the growth of both Gram-negative and Gram-positive bacteria. It was observed that the zone of inhibition is proportional with the amount of Mg doping in ZnO NPs. The results obtained to show the effect of Mg doping in ZnO NPs are illustrated in Table 3. This might be attributed to the reduction in their band gap values. Due to reduction in the band gap, there is a possibility of exciton generation. Overall, this enhances the photocatalyst activities for improved bactericidal activity of Mg-doped ZnO NPs [64]. Furthermore, due to the various surface-interface characteristics may have different chemical-physical, adsorption-desorption abilities in the direction towards bacteria, make sure in different antibacterial performances [65].

**Table 3:** Average diameter values of Zones of Inhibition (ZOI) for pure and Mg-doped ZnO NPs (2.5–7.5 M %)

S. No	Samples	Zone of inhibition (mm)		
		Gram-negative strains	Gram-negative strains	Gram-positive strains
		*Proteus*	*Escherichia coli*	*Staphylococcus aureus*
1	ZnO NPs	6 ± 0.7	9 ± 0.7	9 ± 0.6
2	2.5% Mg-ZnO NPs	9 ± 0.4	10 ± 0.4	13 ± 0.4
3	5% Mg-ZnO NPs	12 ± 0.3	14 ± 0.5	16 ± 0.3
4	7.5% Mg-ZnO NPs	15 ± 0.4	16 ± 0.5	19 ± 0.3

The interaction between the NPs and the cell wall of bacteria was changed due to doping of Mg. The growth of *S. aureus* and the other two bacteria was more commendably affected by Mg^2+^-doped ZnO nanostructures compared with pure ZnO NPs. From Table 3, it is noted that Gram-negative and Gram-positive have different inhibition zones. This difference in the antibacterial activity of Mg-doped ZnO nanostructures against Gram-negative and Gram-positive bacterial strains may be due to the difference in cell wall structure of those respective bacteria. It was also reported earlier that various bacterial strains had considerably different infectivity and tolerance levels towards the different agents including antibiotics [66]. Also differences in the antibacterial activity might be due to different particle dissolution.

Basically, the antibacterial efficiency of pure and Mg-doped ZnO NPs is mainly dependent on the increased levels of reactive oxygen species (ROS), mostly hydroxyl radicals (OH) and singlet oxygen [67]. This is mainly due to the enlarged surface area which causes increase in oxygen vacancies as well as the diffusion capacity of the reactant molecules inside the NPs [68]. The reactive oxygen group contains superoxide radical and hydrogen peroxide. Both of them can damage the DNA and cellular protein leading to cell death [69]. Moreover, the presence or addition of the nanostructures on the surface or cytoplasm of the bacteria can cause the disruption of cellular function as well as disorganization of the cell membranes [70]. The doping of Mg with ZnO may lead to the variation in grain size, morphology, and solubility of Zn^2+^ ions. All these factors combined together have a robust impact on the antibacterial activity of ZnO [71, 72]. The results have revealed that Mg-doped ZnO nanostructures will be a promising candidate to be used for potential drug delivery systems to cure some significant infections in the near future.

## Conclusions

To conclude, pure and Mg-doped ZnO structures were successfully synthesized by co-precipitation method. The XRD patterns revealed the wurtzite structure for all the nanosamples and no impurity phase was noted. The maximum crystallite size obtained from XRD was less than 100 nm. FE-SEM studies confirmed that the crystallite size increased with increase in Mg content. The UV-Visible results revealed that absorption underwent a redshift with Mg into ZnO as compared to pure ZnO exhibiting strong quantum confinement effects. Optical band gap energy was found to decrease from 3.36 to 3.04 eV with Mg doping, resulting in the increment in their crystallite size as a result of Mg doping. PL results confirmed the enhanced visible emissions with Mg-doped ZnO leading to the increase in delocalization of electron-hole pairs. Photocatalytic measurements revealed the increase in Mg doping in the ZnO nanoparticles that caused higher photocatalytic activity. The antibacterial activities of the synthesized nanosamples were tested against *E. coli* (Gram-negative), *S. aureus* (Gram-positive bacteria), and *Proteus* (Gram-negative strains).
